# Marine Seagrass Extract of *Thalassia testudinum* Suppresses Colorectal Tumor Growth, Motility and Angiogenesis by Autophagic Stress and Immunogenic Cell Death Pathways

**DOI:** 10.3390/md19020052

**Published:** 2021-01-22

**Authors:** Ivones Hernández-Balmaseda, Idania Rodeiro Guerra, Ken Declerck, José Alfredo Herrera Isidrón, Claudina Pérez-Novo, Guy Van Camp, Olivier De Wever, Kethia González, Mayrel Labrada, Adriana Carr, Geovanni Dantas-Cassali, Diego Carlos dos Reis, Livan Delgado-Roche, Roberto Rafael Nuñez, René Delgado-Hernández, Miguel David Fernández, Miriam T. Paz-Lopes, Wim Vanden Berghe

**Affiliations:** 1 Instituto de Ciencias del Mar (ICIMAR), Calle Loma #14 e/35 y 37, Alturas del Vedado, Plaza de la Revolución, Havana 10400, Cuba; ivones@icimar.cu (I.H.-B.); kethia@icimar.cu (K.G.); ldelgado@liomont.com.mx (L.D.-R.); robertico@icimar.cu (R.R.N.); migueldavid@icimar.cu (M.D.F.); 2 Laboratory of Protein Science, Proteomics and Epigenetic Signaling (PPES) and Integrated Personalized and Precision Oncology Network (IPPON), Department of Biomedical Sciences, Campus Drie Eiken, University of Antwerp, Building S, 4th Floor, Universiteitsplein 1, 2610 Wilrijk, Belgium; ken.declerck90@hotmail.com (K.D.); Claudina.PerezNovo@uantwerpen.be (C.P.-N.); 3 Instituto de Ciencia y Tecnología de Materiales (IMRE), Universidad de la Habana, Zapata y G, Vedado, Plaza de la Revolución, Havana 10400, Cuba; jose@imre.uh.cu; 4 Center of Medical Genetics, University of Antwerp and Antwerp University Hospital, Prins Boudewijnlaan 43, 2650 Edegem, Belgium; guy.vancamp@uantwerpen.be; 5 Laboratory of Experimental Cancer Research, Department of Radiation Oncology and Experimental Cancer Research, Cancer Research Institute Ghent (CRIG), UZ-Gent, 9000 Gent, Belgium; olivier.dewever@ugent.be; 6 Center of Molecular Immunology, Calle 17, Atabey, Playa, Havana 11300, Cuba; mayrel@cim.sld.cu (M.L.); adriana@cim.sld.cu (A.C.); 7 Institute of Biological Sciences (ICB), Federal University of Minas Gerais (UFMG), Belo Horizonte 31207-901, Brazil; cassalig@icb.ufmg.br (G.D.-C.); dcarlosreis@yahoo.com.br (D.C.d.R.); mtpl@icb.ufmg.br (M.T.P.-L.); 8 Instituto de Farmacia y Alimentos (IFAL), Universidad de La Habana, (UH), Ave. 23 # 21425 Entre 214 and 222, La Coronela, La Lisa, Havana 13600, Cuba; rdelgado@ifal.uh.cu; 9 Facultad de Ciencias Naturales y Agropecuarias, Universidad de Santader (UDES), Bucaramanga 680002, Colombia

**Keywords:** *Thalassia testudinum*, cytotoxicity, antitumor, anti-angiogenic, gene expression

## Abstract

Marine plants have become an inexhaustible reservoir of new phytopharmaceuticals for cancer treatment. We demonstrate in vitro/in vivo antitumor efficacy of a standardized polyphenol extract from the marine angiosperm *Thalassia testudinum* (TTE) in colon tumor cell lines (RKO, SW480, and CT26) and a syngeneic allograft murine colorectal cancer model. MTT assays revealed a dose-dependent decrease of cell viability of RKO, CT26, and SW480 cells upon TTE treatment with IC_50_ values of, respectively, 175, 115, and 60 μg/mL. Furthermore, TTE significantly prevented basal and bFGF-induced angiogenesis in the chicken chorioallantoic membrane angiogenesis assay. In addition, TTE suppressed bFGF-induced migration of endothelial cells in a wound closure assay. Finally, TTE treatment abrogated CT26 colorectal cancer growth and increased overall organism survival in a syngeneic murine allograft model. Corresponding transcriptome profiling and pathway analysis allowed for the identification of the mechanism of action for the antitumor effects of TTE. In line with our in vitro/in vivo results, TTE treatment triggers ATF4-P53-NFκB specific gene expression and autophagy stress pathways. This results in suppression of colon cancer cell growth, cell motility, and angiogenesis pathways in vitro and in addition promotes antitumor immunogenic cell death in vivo.

## 1. Introduction

Colorectal cancer (CRC) is considered one of the most commonly diagnosed malignancies and a leading cause of cancer death worldwide [1]. The last Cuban Health Report indicated that CRC is the third cause of mortality and its incidence ranked fifth, among all cancers [2]. A high number of patients are detected at middle- or late-stage CRC, which results in difficult clinical management and less favorable prognoses. In addition, the treatments fail due to drug resistance, tumor relapse, and/or high adverse toxicity. Thus, it is an urgent need for the development of new anticancer drugs.

There is a renewed interest in plant phytochemicals for cancer treatment [3]. The public World Health Organization (WHO) global report on traditional and complementary medicine indicates that in developing countries, 80% of people rely on plant-derived medicines for health care [4]. Interestingly, besides traditional medicinal plants, marine floras have also become a source of bioactive phytochemicals with antioxidant, immunomodulatory, and antitumor activities [5,6].

Seagrasses are marine angiosperms rich in secondary metabolites, particularly, phenolic compounds [7]. They cover about 50% of the Cuban coasts, with six species reported and *Thalassia testudinum* K.D. Koenig being the most dominant [8]. One aqueous-ethanolic extract has been obtained from the *T. testudinum* (TTE) leaves harvested at Havana coasts. Regalado et al. identified that polyphenols (29.5 ± 1.2%) are the major compound present in this extract [9]. Its major constituents are known [9,10] and standardized to chrysoeriol-7-*O*-β-D-glucopyranosyl-2″-sulfhate (thalassiolin B) content; the main compound is responsible for the antioxidant effects of TTE [9,11]. Apigenin, apigenin 7-*O*-β-D-glucopyranosyl-2″-sulfhate (thalassiolin C), apigenin 7-*O*-β-D-glucopyranoside, chrysoeriol, chrysoeriol 7-*O*-β-D-glucopyranoside, dihydroxy-3′,4′-dimethoxyflavone 7-*O*-β-D-glucopyranoside, and luteolin-3′-sulphate, were present in the extract. Proanthocyanidins were also detected in relevant concentrations (21.0 ± 2.3%) in TTE [9]. A broad spectrum of bioactivities has been reported for the proanthocyanidins glucopyranosyl derivates and their aglycon structures (chrysoeriol, luteolin, and apigenin), including antitumor properties [12,13,14]. Recently, our group showed that TTE inhibits the cell viability of different tumor cells, but not of normal (non-tumor) cells [15]. Taken together, our results show the cytotoxic activity of the polyphenolic fraction of TTE and the thalassiolin B, which it is associated with reactive oxygen species (ROS) generation and pro-apoptotic effects in HCT15, a human colorectal cancer cell line. Furthermore, the antitumor activity of the polyphenolic fraction was demonstrated in a xenograft mice model [16], suggesting that this marine plant holds promise as a rich natural resource of antitumor phytochemicals.

Pharmacological studies already confirmed the strong antioxidant and anti-inflammatory properties of TTE [11,17,18,19]. Since chronic inflammation and oxidative stress conditions promote aggressive CRC progression and therapy resistance [20], clinical studies recommend the therapeutic use of anti-inflammatory and antioxidant compounds for long-term use in patients diagnosed with different tumor types [21]. In this study, anticancer properties of standardized TTE were further characterized in cell viability assays in colon cancer (RKO, SW480, and CT26) cells. In addition, the suppressive, antitumor effects of TTE on cancer cell migration and angiogenesis were evaluated. Furthermore, in vitro/in vivo transcriptome studies were performed of human colon cancer cells (SW480) and syngeneic CT26 allograft tumors treated with TTE to identify key target genes and pathways responsible for its potent anticancer effects.

## 2. Results

### 2.1. TTE Treatment Elicits Dose-Dependent Cytotoxicity against Colon Cancer Cells

**Figure 1:**
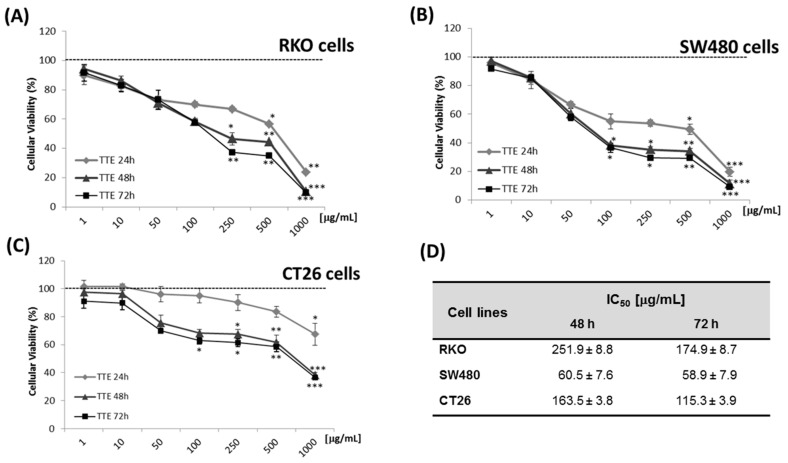
*Thalassia testudinum* extract (TTE) treatment decreased colon cancer cell survival. RKO (**A**), SW480 (**B**), and CT26 (**C**) colon cancer cells were treated for 24, 48, or 72 h with increasing concentrations of TTE (1–1000 µg/mL). Corresponding cell viability was evaluated by MTT assays. Each line plot represents the mean ± SD of three assays * *p* < 0.05, ** *p* < 0.01, and *** *p* < 0.001 compared with vehicle control value. IC_50_ values for the cells are summarized in panel (**D**).

Tumor cell cytotoxic effects of TTE were assessed by MTT viability assays in human RKO, SW480, and mouse CT26 colon cancer cells, in a concentration range of 1 to 1000 μg/mL. Cell viability after exposure to TTE or medium for 24, 48, or 72 h was determined using MTT assay. Treatments with the extract resulted in a significant time- and concentration-dependent decrease of the cell viability in the different type evaluated (Figure 1A–C). The colon cancer cell line SW480 showed the highest sensitivity to the TTE treatment, with IC_50_ values of 174.9, 58.9, and 115.3 μg/mL for RKO, SW480, and CT26 cells, respectively, after 72 of exposure (Figure 1D and Supplementary Figure S1). In contrast, similar doses of TTE did not promote any cytotoxicity in non-tumor cell types (i.e., primary hepatocytes, CHO, VERO, 3T3, MDCK, and BHK-21) [15].

### 2.2. TTE Suppresses Angiogenesis In Ovo in the Chorioallantoic Membrane (CAM) Assay

**Figure 2:**
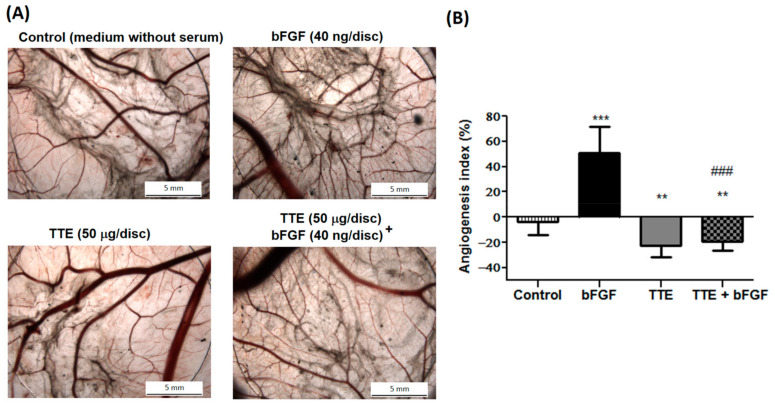
*Thalassia testudinum* extract (TTE) inhibits bFGF-induced neovascularization in the chorioallantoic membrane (CAM) assay. Anti-angiogenic effects of TTE were evaluated in ovo in a chicken CAM assay. (**A**) Representative photographs of blood vessel growth in disks: vehicle control (serum-free medium), bFGF (40 ng/disc), TTE (50 μg/disc) or a combination thereof. (**B**) The $\mathrm{Angiogenic}\;\mathrm{index}\;=\;\left(t\;-\;c\right)/c$ was calculated as described in Section 4. The bar plot graph (mean ± SD) indicates angiogenic index (%) of CAMs. Seven eggs were tested per each condition. Mann–Whitney U-test, ** *p* < 0.01 and *** *p* < 0.001 statistically different from vehicle control, ^###^ *p* < 0.001 different from bFGF exposure.

Angiogenesis plays a pivotal role in progression of malignant tumors and is an attractive target in cancer therapy to block the supply of oxygen and nutrients necessary for the survival of tumor cells or to prevent a route for metastatic spread of these cancer cells. To investigate possible mechanisms of TTE against neovascularization in vivo, we evaluated potential anti-angiogenic effect of TTE in the CAM assay during development of the chicken embryo. No vascular sprouting could be observed in the CAM assay in control conditions with serum-free medium (Figure 2A). Pro-angiogenic endothelial cell proliferation, migration, and tube formation were significantly stimulated by 50% upon bFGF treatment (40 ng/disc), as measured by the angiogenic index [22]. It should be noted that TTE alone significantly reduced the angiogenesis index (%) as compared to control at non-toxic concentrations or exposure times [15,23]. Therefore, data suggest that basal angiogenesis was indeed inhibited by the extract. Furthermore, TTE (50 µg/disc) treatment was able to completely reverse bFGF-induced blood vessel formation to levels 20% below that of basal growth conditions (Figure 2A,B).

### 2.3. TTE Inhibits Migration of Human EA.hy926 Endothelial Cells

**Figure 3:**
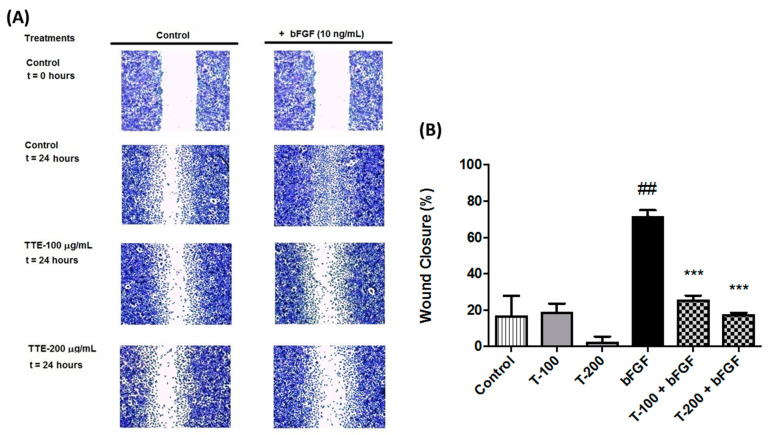
*Thalassia testudinum* extract (TTE) treatment inhibits bFGF-dependent cell migration in the wound healing assay. EA.hy926 cells were treated with vehicle or TTE (0–200 µg/mL) in the presence or absence of bFGF (10 ng/mL). (**A**) The migratory cells were stained with crystal violet and photographed at 0 and 24 h, as indicated. (**B**) The bar plot graph indicates % wound closure of the treatments. Data represent mean ± SD (*n* = 6), Mann–Whitney U-test *** *p* < 0.001 statistically different from bFGF setup, ^##^ *p* < 0.01 different from vehicle control.

During cancer progression, epithelial-mesenchymal transition of cancer cells frequently promotes migration and invasion, eventually leading to cancer cell metastasis [24]. We examined the ability of TTE to directly affect cellular migration of human EA.hy926 endothelial cells using a classic in vitro wound healing model. Confluent monolayers of EA.hy926 cells were scratched with a 200 μL yellow tip. Then, cell migration into the wound from adjacent areas of the monolayer was evaluated at nontoxic concentrations (100 and 200 μg/mL) (Supplementary Figure S2). After 24 h the extent of cellular migration and wound closure of the EA.hy926 cells was measured by crystal violet staining and quantified. As can be observed from Figure 3A,B, both concentrations of TTE significantly suppressed bFGF-induced cell migration and wound healing of EA.hy926 cells.

### 2.4. In Vitro Treatment of SW480 Cells with TTE Promotes Autophagic Gene Expression Changes Involved in Cancer Cell Death, Cell Cycle Arrest and Inhibition of Cell Motility and Invasion

**Figure 4:**
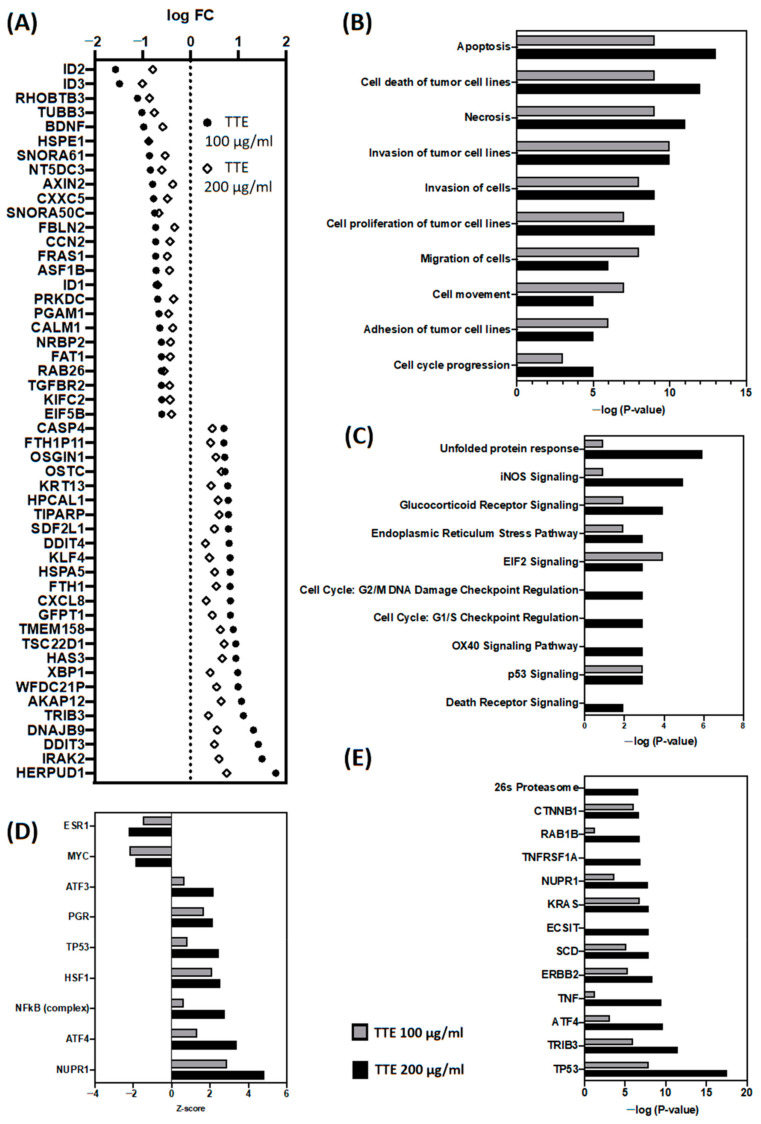
Ingenuity pathway enrichment analysis of differentially expressed genes of SW480 cells treated for 6 h with TTE. (**A**) Gene expression plot of Top 50 up/downregulated genes by SW480 treatment (100–200 µg/mL). (**B**) IPA enrichment analysis of disease functions. (**C**) IPA enrichment analysis of canonical signaling pathways. (**D**) IPA enrichment analysis of transcription factor motifs. (**E**) IPA enrichment analysis of upstream regulators.

To further characterize possible anticancer mechanisms of TTE treatment at the molecular level, we next measured genome-wide transcriptome changes in the most sensitive colon cancer cell line, SW480, following a 6 h treatment with 100 or 200 µg/mL TTE. Using a cutoff expression fold change of ≥1.25 of treated vs. control cells, the R-package “imam” identified 290 up- vs. 254 downregulated or 510 up- vs. 457 downregulated genes following, respectively, 100 or 200 µg/mL TTE treatment of the SW480 cells, of which 142 genes were up- and downregulated by both treatments in a similar, dose-dependent manner (Figure 4A, Supplementary Table S1). Bioinformatic analysis of differentially expressed genes by Ingenuity Pathway Analysis (IPA) revealed that most of the significant gene expression changes in biological pathways related to cell death (apoptosis and necrosis), metastasis (invasion, motility, cell adhesion, and cell movement), and cell proliferation (cell cycle) (Figure 4B and Supplementary Figure S3), in line with our results showing reduced survival, decreased cell proliferation, and suppressed cellular migration-invasion properties in vitro (Figure 1, Figure 2 and Figure 3). More particularly, TTE treatment strongly promoted unfolded protein stress, nitrosative stress (NO), and endoplasmic reticulum stress, which all trigger autophagy gene expression (Figure 4A–C). Finally, analysis of TTE responsive genes identified master regulators of autophagy, including multiple transcription factors P53, ATF4, NFκB, heat shock factor (HSF)1, Nuclear protein (NUPR)-1, (the stress (pseudo)kinase tribble (TRIB), NUPR1, and various hormone receptors (NR3C1, ESR1, and PGR) (Figure 4D–E).

### 2.5. TTE Reduces Tumor Growth and Increases Overall Organism Survival of the Syngeneic Allograft Murine CT26 Colorectal Cancer Model

**Figure 5:**
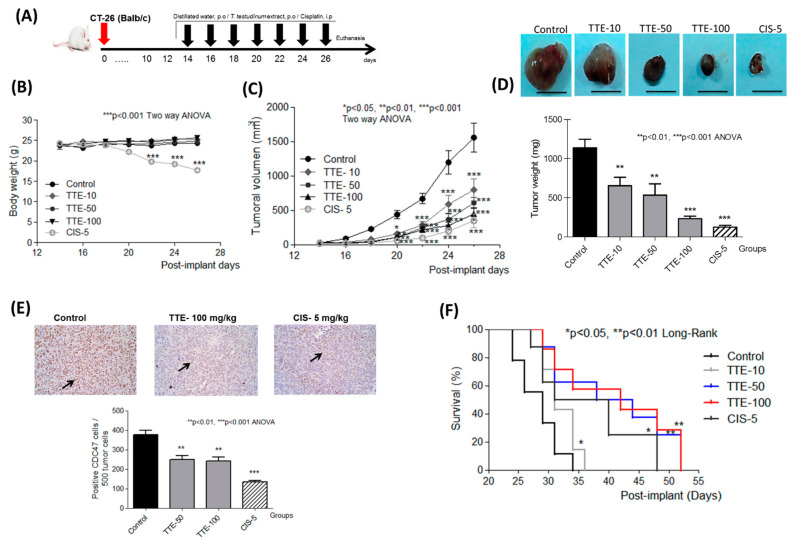
*Thalassia testudinum* extract (TTE) inhibits tumor growth in an ectopic model of syngeneic subcutaneous transplantation of murine colon carcinoma CT26 cells. (**A**) The cells (1 × 10^5^) were injected subcutaneously into 6–8 weeks-old Balb/c mice. After 12 days, animals were orally treated for 14 days with TTE (10, 50, or 100 mg/kg) or intraperitoneally with cisplatin (5 mg/kg) as schematically summarized. (**B**) Body weight changes by treatments. (**C**,**D**) Mean ± SEM of tumor volume and weight. (**E**) Immunohistochemical staining of tumors to CDC47 monoclonal antibody. (**F**) Survival rate. Statistically significant differences were determined by two-way ANOVA * *p* < 0.05; ** *p* < 0.01; *** *p* < 0.001.

To evaluate whether TTE has comparable antitumor effects in vivo, CT26 cells were inoculated subcutaneously into the flanks of Balb/c mice. When tumor volume was higher than 30 mm^3^, the animals were treated daily with the TTE (10, 50, and 100 mg/kg) by oral gavage for two weeks. The group treated with cisplatin received an intraperitoneal dose (5 mg/kg) every five days (positive control group) (Figure 5A). No signs of toxicity were observed during the experience for treated groups with TTE. In contrast, piloerection, decrease in the motor activity, and loss of body weight were observed in mice exposed to cisplatin (Figure 5B). A normal tendency to increase body weight was observed in the negative control and the groups treated with TTE in contrast to that of cisplatin-treated mice (Table 1).

We observed a dose-dependent reduction of tumor growth with TTE treatments, as demonstrated in the significant decrease of tumor volume and weight (Figure 5C,D). TTE treatments showed an antitumor effect with a maximal reduction in tumor size of 70% at 100 mg/kg TTE. Overall, the reference cisplatin cancer treatment (5 mg/kg) revealed the strongest antitumor effect (approximately 78% tumor size reduction) (Table 1). Immunohistochemical staining intensity of the cell proliferation marker CDC47 showed that TTE- and cisplatin-dependent reduction of tumor volume corresponded with decreased immunohistochemical staining intensity of CDC47 (Figure 5E). Moreover, TTE treatment significantly increased organism survival (Figure 5F).

In addition, lipid peroxidation and protein damage, measured by different oxidative stress biomarkers, were significantly lower in TTE groups when compared with that of the negative control. Levels of glutathione and the enzymatic activity of SOD and CAT were higher (*p* < 0.05) in mice receiving doses of 50 and 100 mg/kg of TTE with respect to that of controls (Table 2). As was expected, cisplatin treatment increased lipid peroxidation and decreased the antioxidant enzyme activities.

### 2.6. In Vivo Treatment of the Syngeneic Allograft Model of Murine CT26 Colon Cancer with TTE Triggers a ATF4-P53-NFκB-Specific Gene Response Towards Antitumor Immunogenic Cell Death

**Figure 6:**
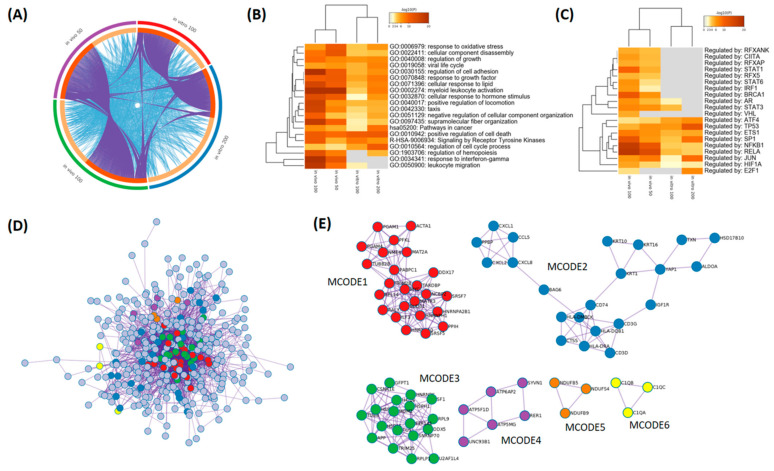
Systems level metascape analysis of transcriptome profiles of *Thalassia testudinum* extract (TTE) treatment in vitro/in vivo. (**A**) The Circos plot shows how genes from the different input gene lists of in vitro/in vivo setups overlap. On the outside, the arc represents the identity of each gene list. On the inside, the orange color represents the genes that appear in multiple lists, and the light orange color represents genes that are unique to that gene list. Purple lines link the same genes shared by multiple gene lists. Blue lines link the genes that fall into the same ontology term (the term has to be statistically significantly enriched). The greater the number of purple links and the longer the dark orange arcs, the greater is the overlap among the input gene lists. Blue links indicate the amount of functional overlap among the input gene lists. (**B**) Metascape enrichment analysis of statistically enriched ontology terms (GO/KEGG terms, canonical pathways, and hall mark gene sets). (**C**) Metascape enrichment analysis of all statistically enriched TF-target interaction networks. (**D**) All protein–protein interactions (PPI) among all input gene lists were extracted from the PPI data source and formed a PPI network. (**E**) GO enrichment analysis was applied to the network to assign biological “meanings” of sub-protein networks. GO enrichment analysis was applied to each MCODE network to assign “meanings” to the network component, where the top three best *p*-value terms were retained. MCODE components were identified from the merged network. Each MCODE network is assigned a unique color.

To confirm the in vitro mechanism of action of TTE in vivo in tumor samples (Figure 5D) after 14 days of TTE treatment, we measured again the corresponding gene expression changes. Corresponding volcano plots revealed multiple significant gene expression changes (fold change cut-off of 1.25) of both TTE treatments (Supplementary Figure S4), which were finally crossc-ompared with in vitro gene expression profiles by Metascape systems biology freeware (https://metascape.org/) [25] (Supplementary Tables S2 and S3). As can be observed in the circos plot (Figure 6A), we observed limited gene overlap between setups, as is common in meta-analysis due to the variations in the experimental approaches. However, in the heatmap representations (Figure 6B), we see a lot more functional overlap, as these studies probably captured different parts of the same biological processes in vitro/in vivo, related to cell death, angiogenesis, cell growth, and cell adhesion, in addition to oxidative stress, receptor tyrosine kinase, and hormone-signaling pathways (Supplementary Table S4 and Supplementary Figure S5). Interestingly, in the syngeneic CT26 colon cancer allograft mouse model we observed additional involvement of IFNγ signaling and leukocyte activation-migration in the tumor immune micro-environment, which could not be addressed in the in vitro treatment setup (Figure 6B). Furthermore, similar to our in vitro experiment, we also identified significant enrichment of p53, ATF4, and NFκB transcription factor motifs in TTE-responsive genes in vivo, whereas the involvement of NFκB target genes seemed to be increased in vivo (Figure 6C). Finally, protein–protein interaction analysis of all TTE-responsive target genes by Metascape analysis identified six protein interaction subnetworks (Figure 6D) involved in RNA splicing (MCODE1), TCR-ZAP70-PD-1 immune signaling (MCODE2), RNA-metabolism (MCODE3), mitochondrial respiration-biogenesis (MCODE4-5), and innate immunity complement complex regulation (MCODE6), which all contribute to myeloid leukocyte activation and immunogenic cell death pathways (Figure 6E and Supplementary Table S5).

## 3. Discussion

The marine environment represents a huge reservoir of novel bioactive metabolites with diverse groups of chemical structures with therapeutic and biotechnological potential [26,27]. The discovery and development of marine drugs against cancer has been extremely rewarding with significant scientific gains, such as the discovery of new anticancer mechanisms of action as well as novel molecular targets [28,29].

In the present study, we applied transcriptome profiling and systems biology approaches to further characterize in vitro/in vivo antitumor activities of a standardized hydroethanolic extract from leaves of the marine angiosperm *Thalassia testudinum* growing on the coasts of Cuba [9,10]. In line with our previous results, time- and dose-dependent loss of cell viability was observed in the colon cancer cell lines (RKO, SW480, and CT26), whereas non-tumor cells remained unaffected [15,16]. Among all tested colon cancer cell types, SW480 was identified as the most sensitive to TTE treatment with IC_50_ values of 60 µg/mL. In line with our results, RKO cells were found to be more aggressive colorectal cancer cells than were SW480 cells in migration-motility assays due to their stronger mesenchymal stem cell and less epithelial phenotype, which may make them less vulnerable (or more resistant) to TTE treatment [30,31].

To date, various anticancer flavonoids have been identified in TTE, such as apigenin, luteolin, chrysoeriol-7-*O*-β-D-glucopyranoside, and thalassiolin B [9,12,13,16,32,33,34]. Similar to TTE, apigenin treatment of SW480 cells shows comparable cell cytotoxicity (IC_50_ value 40 µM) [35,36]. In addition, chrysoeriol-7-*O*-β-D-glucopyranoside and luteolin were found to be effective antitumor bioactive molecules against colon cancer cells [37]. Recently, we showed that thalassiolin B and the polyphenolic fraction isolated from TTE both mimicked induced ROS production and tumor regression in a xenograft colorectal cancer model [16]. Accordingly, antitumor efficacy of TTE could be attributed to the presence of thalassiolin B, apigenin, chrysoeriol-7-*O*-β-D-glucopyranoside, and luteolin in this mixture [16,35,36,37,38].

Upon transcriptome profiling of TTE-treated SW480 cells, we found that TTE triggers multiple stress pathways (unfolded protein stress, endoplasmic reticulum stress, nitrosative stress, and DNA damage) that exceed the stress tolerance of colon cancer cells. Accordingly, cell cycle arrest and autophagy rescue gene responses by ATF4-P53-NFkB transcription factors fail to restore homeostasis upon too many stress insults. Additional gene expression and pathway enrichment analysis also revealed inhibition of cell proliferation-motility-invasion-angiogenesis programs, which together with loss of autophagy damage control will progress towards colon cancer cell death. Today, several studies show that various phytochemicals, including TTE constituents such as luteolin, apigenin, and chrysoeriol can efficiently modulate autophagy-dependent cancer cell death [12,13,32,38,39,40,41,42]. Similarly, emodin (present in Chinese medicinal herbs *Rheum* and *Polygonum*) and capsaicin (present in chili peppers) were shown to trigger NFkB/TRIB3-dependent autophagic cell death in different cancer cell types [43,44,45].

Moreover, our in vitro cell viability, wound healing, and CAM assay results (Figure 1, Figure 2 and Figure 3) further support our gene expression pathway analysis, showing decreased cell viability, tumor cell migration, and angiogenesis in the presence of TTE. Apigenin and luteolin could be responsible for these TTE treatment effects, since both compounds have been reported to inhibit vasculogenesis in the CAM assay and to decrease cell migration (Supplementary Table S6) [33,35,46,47,48]. However, despite the enriched presence of these bioactive phytochemical constituents in the extract, other components cannot be excluded. Finally, TTE treatment also reduced tumor growth in vivo and increased overall organism survival of a syngeneic allograft murine CT26 colon tumor model. At the transcriptome level, we observed nice overlap of TTE-responsive antitumor stress response and cell growth pathways in vitro/in vivo modulated by ATF4-P53-NFκB gene expression programs. Of particular interest, in vivo, we observed an additional role for immune signaling pathways (IFNγ, PD-1, and ZAP70) and myeloid leukocyte activation-migration into the tumor microenvironment (TME) to promote immunogenic cell death [43]. Tumor cells frequently produce soluble factors that favor myelopoiesis and recruitment of myeloid cells to the TME. Consequently, the TME of many cancer types is characterized by high infiltration of monocytes, macrophages, dendritic cells, and granulocytes [49]. Experimental and clinical studies show that most myeloid cells are kept in an immature state in the TME by tumor secreted factors that support cancer initiation and progression, amongst other activities, by enabling immune evasion, tumor cell survival, proliferation, migration, and metastasis. For example myeloid cells support immune evasion in cancer through EGFR/MAPK-dependent regulation of PD-L1 expression in tumor cells [50]. Interestingly, PD-1, a T cell checkpoint receptor and target of cancer immunotherapy, is also expressed on myeloid cells, which orchestrate immune checkpoint blockades [51]. As such, strategies to deplete myeloid cells or redirect their function in the TME hold promise to overcome resistance to current cancer therapies. For example, targeted deletion of PD-1 in myeloid cells was already shown to induce antitumor immunity [52]. Similar to our results, therapeutic modulation of myeloid polarization through the IFN-γR/STAT1 signaling axis was found to enhance antitumor immune function by activating tumor infiltrating myeloid cells, regulating PD-L1 expression, and promoting immunogenic cell death [53,54,55,56,57,58]. New studies indicate that autophagy in addition to its cell-autonomous antitumorigenic functions inhibits cancer development by orchestrating inflammation and immunity. While attenuating tumor-promoting inflammation, autophagy enhances the processing and presentation of tumor antigens and thereby stimulates antitumor immunity. Although cancer cells can escape immune-surveillance by tuning down autophagy, phytochemical agents with immunogenic and autophagy-promoting properties may enhance antitumor immunity by inducing autophagic cell death [54]. For apigenin, luteolin, and chrysoeriol, immunogenic and autophagy-promoting properties have already been reported (Supplementary Table S6) [32,33,38,39,59]. In addition, we previously found that treatment with another purified TTE constituent (Thalassiolin B) fully mimicked ROS production and tumor regression by polyphenolic fraction treatment in a xenograft colorectal cancer model [16]. Thus, the antitumor efficacy of TTE could be attributed to the presence of multiple bioactive compounds, which exhibit a broad spectrum of therapeutic properties in vitro/in vivo.

## 4. Materials and Methods

### 4.1. Plant Material

*Thalassia testudinum* Banks & Sol. ex K. D. Koenig (Hydrocharitaceae) leaves were collected in March 2016 from “Guanabo” beach (22°05′45″ N, 82°27′15″ W). The plant was authenticated by Dr. Beatriz Martínez Daranas (Center of Marine Research, Havana University, Cuba) with a standard sample deposited in the herbarium of the Cuban National Aquarium (No. IdO40).

The extract was prepared as described previously [10]. Briefly, whole dry and ground *Thalassia testudinum* leaves were continuously extracted (three times) with EtOH-H_2_O (50:50, v:v) for 24 h at room temperature. The sample was filtered and concentrated under reduced pressure and temperature (40 °C) to yield the extract (TTE). TTE composition was standardized according to the percentage of thalassiolin B (within the range 5.8 ± 0.3%), and contained phenolic components (29.5 ± 1.2%), flavonoids (4.6 ± 0.2%), and proanthocyanidins (21.0 ± 2.3%) [9,10]. For in vitro assays, stock solutions of the extract (1 mg/mL) were freshly prepared in culture medium and diluted to achieve the final concentrations tested in each experimental series. Control cells (only medium-treated cells) were included in all experiments. For in vivo studies, the extract was prepared in distilled water.

### 4.2. Cell Lines and Cell Culture

Human colon cancer cell lines (RKO and SW480), the murine adenocarcinoma of colon cell line (CT26), and the human endothelial cell type (EA.hy926, a hybrid of HUVEC with human adenocarcinoma cells) were purchased from the American Type Culture Collection (ATCC, Manassas, VA USA). Cells were grown in Dulbecco’s Modified Eagle Medium (DMEM) and the culture mediums were supplemented with 10% Fetal Bovine Serum, 2 mM L-glutamine, 50 IU/mL penicillin, and 50 μg/mL streptomycin. Cells were incubated at 37 °C in humidified atmosphere containing 5% CO_2_. All cell culture reagents were purchased from Life Technologies (Praisley, UK).

For the syngeneic CT26 allograft model [60], cells were harvested from subconfluent cultures (70–85%) by trypsinization (0.05% trypsin, 0.02% EDTA) at the day of implantation and they were resuspended in buffer phosphate solution (PBS).

### 4.3. MTT Assay

Cell viability was assessed by using 3-(4,5-dimethylthiozol-2-yl)-2,5-diphenyltetrazolium bromide (MTT) (Sigma Aldrich, St. Louis, MO, USA) as previously described [61]. Cells were exposed to TTE (1–1000 μg/mL) for 24, 48, and 72 h. After treatments, MTT was added to wells and the plates were incubated at 37 °C for 4 h and the formazan crystals that formed were extracted with DMSO. The absorbance was read on a microplate reader (Bio Rad Model 3550-UV, Tokyo, Japan) at 595 nm. Experiments were performed at least three times. The concentration of extract required for 50% inhibition of the cell viability (IC_50_) was determined by plotting the percentage of cell viability versus log concentration.

### 4.4. Chorioallantoic Membrane (CAM) Angiogenesis Assay

This assay was performed as described [62]. Briefly, fertilized eggs were incubated for 3 days at 37 °C and humidity of 48%. The eggs were incubated until day 10, prior to application of the extract. TTE-50 μg/disc, bFGF-40 ng/disc, TTE-50 μg/disc plus bFGF-40 ng/disc, and control (medium culture) were poured onto separate discs (12 mm diameter), under sterile conditions for 72 h. Test discs probed with recombinant human bFGF (Peprotech) served as a control for angiogenesis stimulation. At the end of the assay, the CAM, the area of the discs included, was placed in a petri dish with 10% buffered formalin. The plastic discs were removed and phase-contrast pictures of the area were taken. The vascular index was measured as described previously [22,62]. Vascular intersections on a grid containing three concentric circles (6, 8, and 10 mm diameter) were counted. The $\mathrm{Angiogenic}\;\mathrm{index}\;=\;\left(t-c\right)/c$, where t is the number of intersections in the area covered by the test disc and c is the number of intersections in the area covered by the control disc in the same egg.

### 4.5. Wound Healing Migration Assay

A wound healing assay was carried out to determine the migration ability of EAhy926 cells as previously described [63]. A sterilized 200 μL pipette tip was used to generate a scratch ‘‘wound’’ across the cell monolayer and the wound was washed with PBS. The cells were cultured containing concentrations of TTE (100 and 200 μg/mL) for 24 h at 37 °C. Control cells were cultured in medium alone. All cells were stimulated with bFGF (10 ng/mL) to evaluate the effects on migration induction. At the end, cells were washed, fixed with absolute methanol, and stained with crystal violet (2%) [64]. Images from the region adjacent to the wound into which cells migrated were taken with a camera attached to bright-field microscope (Olympus, Tokyo, Japan). Three randomly selected views along the scraped line were photographed on wells at 100×. A reduction in the wounded area indicates a sign of migration. Wound closure was quantitated using the ImageJ software for stained cells and defined as % of area covered after 24 h and compared to time zero [65]. The experiments were performed in triplicate.

### 4.6. RNA Extraction and Illumina Microarray Processing

Total RNA of untreated control or exposure to TTE-treated (100 and 200 μg/mL) SW480 cells was isolated by using an RNeasy Mini Kit (Qiagen, Venlo, Netherlands) according to the manufacturer’s protocol. Following RNA extraction and concentration measurement (NanoDrop 1000, Thermo Scientific, Waltham, MA, USA), quality control was measured on a Bio-Rad experion (Bio-Rad, Hercules, CA, USA). First, 500 ng of total RNA was amplified using the Illumina Total Prep RNA Amplification kit (Life Technologies, Carlsbad, CA, USA). Briefly, RNA was reverse transcribed using T7 oligo(dT) primers, after which biotinylated cRNA was synthesized through an in vitro transcription reaction. Then, 750 ng of amplified cRNA was hybridized to a corresponding array of a HumanHT12 beadchip (Illumina, San Diego, CA, USA). The beadchip was incubated for 18 h at 58 °C in a hybridization oven under continuous rocking. After several consecutive washing steps (see manufacturer’s protocol), bead intensities were read on an Illumina Iscan.

### 4.7. Ectopic Subcutaneous Syngeneic CT26 Colon Cancer Allograft Mouse Model

The studies were carried out in accordance with European regulations on animal protection (Directive 86/609) and the Guide for the Care and Use of Laboratory Animals, US National Institute of Health (NIH Publication № 85–23, 1996). The protocol was approved by the Institutional Animal Care and Ethical Committee, Institute of Marine Sciences (ICIMAR), Havana, Cuba (Protocol number 1501, approved: 12 January 2015). Male Balb/c mice (6–8 weeks of age, 18–22 g) were purchased at the National Laboratory Animal Production Center, Havana, Cuba. They were supplied with water and food ad libitum, under an environment controlled for temperature (22 ± 2 °C), humidity (77 ± 3%), and cycles of 12 h light/dark. The animals were inoculated subcutaneously on the right dorsal side with 1 × 10^5^ CT26 cells/mouse in 100 μL of PBS. Once tumors grew up to 30 mm^3^, mice were randomly divided into five groups (10 animals/group) as follows: distilled water (negative control), TTE 10, 50, and 100 mg/kg, cisplatin 5 mg/kg (positive control). TTE was administered daily intragastrically for 14 days and cisplatin every 5 days by intraperitoneal injections. The tumor growth and size were measured by length and width with calipers every 2 days. The tumor volume was calculated $(V,{\;\mathrm{mm}}^{3}=\mathrm{length}\;\times {\;\mathrm{width}}^{2}\times \;0.5$). At the end, all mice were sacrificed; samples of blood and tumors were taken for the analysis. Percentages of inhibition in growth of the tumor were calculated using the following equation: $\mathrm{Inhibition}\;\left(\%\right)=1-T/C*100$, where T and C are the tumor volumes at the end point for each group.

#### Survival Rate

Another experiment with the same setting was performed to determinate the survival time of the animals. The dates of death of the animals were recorded daily to establish the survival curve. Based on the standard animal protocol, the mice with tumors exceeding 2000 mm^3^ and exhibiting signs of serious illness (i.e., tumor ulceration) were sacrificed.

### 4.8. Statistical Analysis

Statistical analyses were performed with GraphPad Prism 5.0 (GraphPad, La Jolla, CA, USA). Significant differences were identified using the nonparametric Kruskal–Wallis test followed by post-hoc Dunn’s multiple comparison tests for the MTT data. The results of CAM and migration assays were examined using the Mann–Whitney U-test. Body weight, tumor weight and size, and immunohistochemical and oxidative stress biomarkers were identified using an ANOVA test followed by post-hoc Dunnet’s multiple comparison tests. The level of statistical significance was set to * *p* < 0.05, ** *p* < 0.01, and *** *p* < 0.001 for all analyses.

For the microarray, raw data intensities generated were read in R and quantile normalized using the “limma” package [66]. Pathway analysis of SW480 cells was performed using the Ingenuity Pathway Knowledge Base (Ingenuity^®^ Systems, www.ingenuity.com, Redwood City, CA, USA). A fold change cut-off of 1.25 was set to identify genes whose expression was differentially regulated. Fischer’s exact test was used to calculate a *p*-value determining the probability that each biological function and/or disease assigned to that data set was due to chance alone. Metascape systems biology freeware (https://metascape.org/) was used for correlating the transcriptomic profiles of the in vitro/in vivo data [25]. The Circos plot visualization shows how genes from different input gene lists overlap. Heatmaps show Metascape enrichment analysis of all statistically enriched ontology terms (GO/KEGG terms, canonical pathways, and hall mark gene sets). Accumulative hypergeometric *p*-values and enrichment factors were calculated and used for filtering. Remaining significant terms were then hierarchically clustered into a tree dendrogram based on Kappa-statistical similarities among their gene memberships. The term with the best *p*-value is selected within each cluster as a representative term to be displayed in a hierarchical tree dendrogram. The heatmap cells are colored by their *p*-values (see color legend). Along the same line, Metascape enrichment analysis of all statistically enriched TF-target interaction networks was determined by the TRRUST database [67]. Protein–protein interactions (PPI) among all input gene lists were extracted from the PPI data source to form a PPI network. GO enrichment analysis was applied to the network to assign biological “meanings” of sub-protein networks. GO enrichment analysis was applied to each MCODE network to assign “meanings” to the network component, where the top three best *p*-value terms were retained. MCODE components were identified from the merged network. Each MCODE network was assigned a unique color.

## 5. Conclusions

In summary, the results show the potent antitumor effects of *T. testudinum* extract in colorectal cancer cells and in a syngeneic murine colorectal cancer model. In addition, by applying systems biology approaches, a novel mechanism of action is proposed that involves ATF4-P53-NFκB specific gene expression and autophagy stress pathways, which suppress colon cancer cell growth, cell motility, and angiogenesis in vitro and promotes antitumor immunogenic cell death in vivo. The data support the potential use of the *T. testudinum* marine plant as a novel nutraceutical adjuvant in colon cancer (immune) therapy.

## Figures and Tables

**Table 1:** The *T. testudinum* extract effects on corporal weight gain and tumor growth in mice inoculated with CT26 cells.

Groups	Body Weight Gain (g)	Tumoral Volume (mm^3^)	Tumor Growth Rate (mm^3^/day)	Inhibition (%) (1 − T/C) × 100
Control	0.37 ± 0.2	1557.0 ± 206.9	129.40 ± 10.7	-
TTE: 10 mg/kg	1.26 ± 0.3	795.1 ± 162.9 **	65.55 ± 8.4 ***	48.94 ± 10.5
TTE: 50 mg/kg	1.19 ± 0.4	602.7 ± 79.9 ***	47.03 ± 5.1 ***	61.30 ± 5.1
TTE: 100 mg/kg	2.01 ± 0.3 **	476.7 ± 103.7 ***	33.46 ± 3.9 ***	69.39 ± 6.7
CIS: 5 mg/kg	−6.25 ± 0.4 ***	346.0 ± 96.1 ***	24.98 ± 4.2 ***	77.78 ± 6.2

Values represent mean ± SEM, 10 animals per group. Tumor volumes that were measured until the 26th day of injection were regressed, and the slope was taken as the tumor growth rate. Inhibition (%): (1 − T/C) × 100, where the T/C ratio is the tumor volume of treatment group/tumor volume of control group × % at the end time point. ** *p* < 0.01, *** *p* < 0.001 statistically different with respect to control animals (ANOVA, Dunnet’s a posteriori).

**Table 2:** The effects of *T. testudinum* extract on systemic oxidative stress in mice inoculated with CT26 cells.

Groups	Biomarkers				
	MDA (µmol/L)	AOPP (µmol chloramines/L)	GSH (µmol/L)	SOD (U/L)	CAT (U/L)
Control	6.84 ± 0.2	47.40 ± 1.4	36.30 ± 2.7	28.74 ± 1.7	331.6 ± 5.8
TTE-10 mg/kg	6.41 ± 0.3	43.47 ± 2.2	58.52 ± 7.2	34.91 ± 1.2	351.5 ± 7.1
TTE-50 mg/kg	6.18 ± 0.3	22.16 ± 2.0 ***	154.80 ± 7.1 ***	53.99 ± 1.5 ***	476.0 ± 4.6 ***
TTE-100 mg/kg	4.89 ± 0.2 ***	9.93 ± 1.5 ***	171.10 ± 7.9 ***	62.41 ± 1.9 ***	518.2 ± 6.6 ***
CIS-5 mg/kg	7.98 ± 0.4 *	47.49 ± 3.2	31.85 ± 7.4	20.61 ± 3.5 *	289.0 ± 9.9 **

Values represent mean ± SEM of serum biomarkers of oxidative stress. MDA, malondialdehyde; AOPP, advanced oxidation protein products; GSH, reduced glutathione. SOD, superoxide dismutase; CAT, catalase. Serum samples were analyzed by triplicate on two different days. * *p* < 0.05, ** *p* <0.01, *** *p* < 0.001 statistically different with respect to control animals (ANOVA, Dunnet’s a posteriori).

## Supplementary Materials

The following are available online at https://www.mdpi.com/1660-3397/19/2/52/s1, Figure S1: Concentration–response curves of the effect of TTE on the viability of colon cancer cells: (**A**) RKO, (**B**) SW480, and (**C**) CT26. Cell viability was evaluated by MTT assay and IC_50_ values (mean ± SEM) were calculated after 48 h and 72 h of exposure to TTE (1–1000 μg/mL). Figure S2: TTE effects on the viability of endothelial cells. EAhy926 cells were treated with TTE (1–1000 µg/mL) for 24 h. Cell viability was evaluated by MTT assay. Each line plot represents the mean ± SD of three assays. * *p* < 0.05 and ** *p* < 0.01 compared with vehicle control value. Figure S3: Gene expression changes of TTE treatment (i.e., 100 or 200 μg/mL as indicated) involved in apoptosis cell death, vascular tumor growth, and metastasis-proliferation pathways. Figure S4. Volcanoplot of gene expression changes of CT26 tumor samples after 14 days of treatment (TTE, 50 and 100 mg/kg). Figure S5: Heatmap representation of top 20 gene expression changes following 14 days of TTE treatment (50 and 100 mg/kg) and related to cell death, angiogenesis, and cell adhesion-metastasis. Table S1: Differentially expressed genes by TTE in SW480 cells in vitro for IPA analysis. Table S2: Differentially expressed genes by TTE in vivo, input gene lists for Metascape analysis. Table S3: Metascape gene annotation and enrichment analysis of DEG by TTE in vitro/in vivo. Table S4: Metascape GO/KEGG enrichment analysis (Top20–Top100). Table S5: Metascape PPI enrichment analysis: total network versus 6 sub-networks. Table S6: A brief summary of bioactive compounds in a TTE mixture; in vitro and in vivo effects on cancer models.
